# Role of probiotic supplementation in preventing ventilator-associated pneumonia among critically ill patients—a critical umbrella review of meta-analyses of randomized controlled trials

**DOI:** 10.3389/fnut.2025.1719310

**Published:** 2026-02-27

**Authors:** Yan Jiang, Dan Xiao, Jixin Zhou, Fengpei Zhang, Zhiteng Xiong, Qihui Shen, Xiaoyun Xiong

**Affiliations:** 1 Department of Nursing, The Second Affiliated Hospital of Nanchang University, Nanchang, Jiangxi, China; 2 School of Nursing, Jiangxi Medical College, Nanchang University, Nanchang, Jiangxi, China

**Keywords:** critically ill, preventing, probiotics, umbrella review, ventilator-associated pneumonia

## Abstract

**Background:**

In critically ill patients, gut microbiome balance is often disrupted by antibiotics and disease-related stress. Probiotics may strengthen gut barrier function and lower the risk of ventilator-associated pneumonia (VAP), but their effectiveness in mechanically ventilated patients remains unclear. This umbrella review synthesizes evidence from systematic reviews on the association between probiotic therapy and VAP incidence.

**Methods:**

A comprehensive search was conducted in PubMed, Embase, Web of Science, the Cochrane Library, Scopus, and China National Knowledge Infrastructure (CNKI) for systematic reviews published from database inception to July 20, 2025. Data were extracted using a standardized form that had been pilot-tested prior to use. Data were synthesized using both narrative and quantitative approaches. The study protocol was registered in the International Prospective Register of Systematic Reviews (PROSPERO) (registration ID: CRD420251034247).

**Results:**

This umbrella review included 24 meta-analyses of randomized controlled trials (RCTs) involving 92,711 mechanically ventilated critically ill patients. Using a measurement tool to assess systematic reviews, version 2 (AMSTAR 2) tool, the methodological quality varied—a total of 4 were rated critically low, 12 were rated low, 1 was rated moderate, and 9 were rated high. Probiotic supplementation was associated with a reduced risk of VAP [odds ratio (OR) = 0.67, 95% confidence interval (CI): 0.61–0.75; relative risk (RR) = 0.74, 95% CI: 0.69–0.80] and nosocomial infections (OR = 0.81, 95% CI: 0.73–0.90; RR = 0.84, 95% CI: 0.80–0.88). Probiotics showed modest reductions in intensive care unit (ICU) stay [weighted mean difference (WMD) = −1.30 days, 95% CI: −1.59 to–1.02], overall hospital stay (WMD = –1.29 days, 95% CI: −1.79 to −0.79), duration of mechanical ventilation (WMD = –1.64 days, 95% CI: −2.07 to −1.22), and antibiotic use (WMD = –1.26 days, 95% CI: −2.25 to −0.28). The risk of diarrhea decreased based on OR estimates (OR = 0.77, 95% CI: 0.67–0.88), whereas RR estimates did not show a statistically significant difference (RR = 0.98, 95% CI: 0.94–1.01). Probiotic use was associated with a statistically significant reduction in ICU mortality (OR = 0.86, 95% CI: 0.79–0.94; RR = 0.94, 95% CI: 0.90–0.98), whereas hospital mortality was reduced only in RR analyses (RR = 0.92, 95% CI: 0.88–0.97) and not in OR Analyses (OR = 0.92, 95% CI: 0.84–1.01).

**Conclusion:**

Probiotics may offer potential benefits for mechanically ventilated, critically ill patients by reducing infections and improving certain clinical outcomes; however, the overall quality of the available evidence remains insufficient to support definitive conclusions.

**Systematic review registration:**

https://www.crd.york.ac.uk/prospero/display_record.php?ID=CRD420251034247, CRD420251034247.

## Introduction

Critical illness is often accompanied by a reduction in commensal microbiota and an overgrowth of potential pathogens, which increases the risk of sepsis, multiple organ dysfunction syndrome (MODS), ventilator-associated pneumonia (VAP), and hospital-acquired infections. This imbalance markedly elevates morbidity and mortality among patients in intensive care units (ICUs) (1). VAP, defined as pneumonia occurring in patients after more than 48 h of mechanical ventilation (MV), is the second most common hospital-acquired infection among critically ill patients (2, 3). Its incidence rises significantly with longer MV duration and represents a major cause of infection-related mortality (2, 3). VAP not only prolongs ICU and hospital stays but also increases antibiotic use, thereby exacerbating antimicrobial resistance and straining healthcare resources (2, 3). The pathogenesis of VAP is complex, typically involving colonization of the upper aerodigestive tract by pathogens and subsequent aspiration into the lower respiratory tract; infection-related sepsis remains a major global health challenge, with an estimated 21.4 million deaths worldwide in 2021, of which 15.5 million were due to infectious causes (5). These data underscore the need for effective strategies to prevent infections and sepsis in critically ill patients.

Probiotics are live, non-pathogenic microorganisms widely available as commercial supplements, and they have gained increasing attention due to their health benefits. They can be administered alone or in combination with prebiotics—non-digestible carbohydrates that promote the growth or activity of specific microbes (4, 6). Formulations containing both probiotics and prebiotics are termed synbiotics (7). The beneficial effects of probiotics may be attributed to reduced bacterial translocation, stimulation of the commensal gut microbiota growth, and multiple mechanisms that prevent pathogenic overgrowth, including the production of metabolites (e.g., bacteriocins, hydrogen peroxide, lactic acid), nutrient competition, inhibition of pathogen adhesion, and toxin neutralization. They may also promote epithelial cell proliferation (3, 6, 8–13). Accordingly, probiotic therapy has been considered a promising adjunct or alternative to antibiotics in critically ill patients. Several studies and recent meta-analyses have reported that probiotics may provide benefits in ICU patients, such as reducing infection risk, decreasing antibiotic use, lowering the incidence of diarrhea and respiratory infections, and preventing VAP related to loss of protective flora (14–19). However, findings remain inconsistent, leading to conflicting conclusions regarding their role in critical illness. Therefore, we conducted this umbrella systematic review and meta-analysis to synthesize existing evidence on the effects of probiotics on VAP risk in mechanically ventilated critically ill patients and to reconcile inconsistencies across previous meta-analyses.

## Materials and methods

### Search strategy

This systematic review and umbrella meta-analysis (i.e., a meta-analysis of previous meta-analyses) was conducted in accordance with the Preferred Reporting Items for Systematic Reviews and Meta-Analyses (PRISMA) guidelines (20). The study protocol was prospectively registered in the PROSPERO database (CRD420251034247). A comprehensive literature search was performed across PubMed, Embase, Web of Science, Cochrane Database of Systematic Reviews, Scopus, and CNKI from inception to July 2025. The search strategy combined relevant Medical Subject Headings (MeSH) terms and keywords, with language restricted to English and Chinese. The terms included the following: Probiotics (MeSH terms) OR prebiotics (MeSH Terms) OR synbiotics (MeSH Terms) OR Yogurt (MeSH Terms) OR bacillus (Title/Abstract) OR Lactococcus (Title/Abstract) OR Lactobacillus (Title/Abstract) OR bifidobacterial (Title/Abstract) OR bifidobacterium (Title/Abstract) OR Saccharomyces (Title/Abstract) OR Streptococcus (Title/Abstract) OR Culturelle (Title/Abstract) OR *Streptococcus* (Title/Abstract) OR lactic acid bacteria (Title/Abstract) OR yeasts (Title/Abstract) OR pediococcus (Title/Abstract) AND pneumonia, ventilator-associated (MeSH Terms) OR ventilator-associated pneumonia (Title/Abstract) AND meta-analysis (MeSH Terms) OR meta-analyses (Title/Abstract) OR systematic review (Title/Abstract). Detailed search strategies for each database are provided in the supplementary materials. Literature screening and selection were independently performed by two authors (YJ and DX). Additionally, the reference lists of relevant studies were manually checked to identify any potentially eligible articles that may have been missed during the electronic search.

### Inclusion and exclusion criteria

Systematic reviews and meta-analyses examining the effects of probiotic supplementation on outcomes in critically ill patients were considered for inclusion. Outcomes of interest included length of stay in the ICU and hospital, ICU and hospital mortality, duration of mechanical ventilation, duration of antibiotic use, and the incidence of VAP, nosocomial infections, and diarrhea among mechanically ventilated patients. Studies were eligible if they reported effect estimates (ES) with corresponding confidence intervals (CI) and met the methodological requirements of this umbrella meta-analysis. The following types of studies were excluded: *in vitro* or animal studies, case reports, conference abstracts, letters, observational studies, quasi-experimental studies, non-randomized controlled trials (RCTs), and studies with insufficient data or unavailable full texts. The study selection process was independently conducted by two reviewers (YJ and DX), with any disagreements resolved through discussion with a third author (JZ).

In evaluating the effect of probiotics on VAP in critically ill patients, included systematic reviews and meta-analyses did not stratify participants by race, sex, nationality, or geographic region. The population, intervention, comparator, outcome, and study design (PICOS) framework for this study was defined as follows: Population (P): Critically ill adult and pediatric patients receiving mechanical ventilation; Intervention (I): Treatment with probiotics, dosage was not restricted; Comparator (C): Control groups, such as placebo or standard care without probiotic supplementation; Outcomes (O): Incidence of VAP, mortality, ICU and hospital length of stay, duration of mechanical ventilation, and other relevant clinical measures; Study design (S): Randomized controlled trials (RCTs). Only meta-analyses reporting effect estimates and corresponding confidence intervals for the effect of probiotics on VAP in critically ill patients were included in this umbrella review. Studies were excluded if they exclusively involved prebiotics, focused on neonates (participants aged <28 days), were *in vitro* experiments, case reports, conference abstracts, letters, observational or quasi-experimental studies, non-randomized trials, were published in languages other than English or Chinese, or lacked sufficient data or full-text availability.

### Assessment of study overlap

To evaluate the extent of overlap among primary studies included in the meta-analyses, we constructed a citation matrix and calculated the Corrected Covered Area (CCA) index, following the methodological recommendations outlined in the PRIOR statement (50), the Cochrane Handbook (51), and other relevant guidelines (52).


$$\mathrm{CCA}=\frac{N-r}{(r\times c)-r}$$

where N represents the total number of publications, including duplicates, that is, the total number of marked cells in the citation matrix; r is the number of rows, corresponding to the number of indexed publications; and c is the number of columns, corresponding to the number of systematic reviews. The resulting CCA ranges from 0 to 100%.

Interpretation thresholds were applied as follows: ≤5% (slight overlap), 6–10% (moderate overlap), 11–15% (high overlap), and ≥15% very high overlap. The CCA for the present review was 12.1%, indicating a high level of overlap. The complete citation matrix is presented in Table 1.

**Table 1:** Citation matrix of included meta-analyses.

Original research meta-analysis	Zhang et al. (2019) (22)	Bo et al. (2014) (8)	Siempos et al. (2010) (9)	Cheema et al. (2022) (23)	Fan et al. (2019) (24)	Gu et al. (2012) (4)	Liu et al. (2012) (12)	Manzanares et al. (2016) (1)	Sun et al. (2022) (25)	Wang et al. (2013) (26)	Su et al. (2020) (13)	Petrof et al. (2020) (27)	Lee et al. (2023) (28)	Ji et al. (2021) (2)	Chen et al. (2018) (29)	Batra et al. (2020) (3)	Li et al. (2021) (30)	Weng et al. (2017) (31)	Wang et al. (2022) (32)	Zhao et al. (2021) (33)	Sharif et al. (2022) (34)	Lan et al. (2022) (35)	Li et al. (2022) (36)	Song et al. (2022) (37)
Forestier (2008) (54)	√	√	√	√	√	√	√	√	√	√	√	√	√	√	√		√	√	√	√	√	√	√	√
Giamarellos-Bourboulis (2009) (55)	√	√		√	√	√	√		√	√	√		√	√		√	√	√	√	√	√		√	√
Knight (2009) (56)	√	√	√	√	√	√	√	√	√	√	√	√	√	√	√	√	√	√	√	√	√	√	√	√
Morrow (2010) (15)	√	√		√	√	√	√	√	√	√	√	√	√	√	√	√	√	√	√	√	√	√	√	√
Barraud (2010) (57)	√	√		√	√	√	√	√	√	√	√	√	√	√	√	√	√	√	√	√	√	√	√	√
Tan (2011) (58)	√	√		√	√		√	√	√		√	√	√	√	√	√	√	√	√	√		√	√	√
Rongrungruang (2015) (59)	√			√	√			√	√		√		√	√	√		√	√	√	√	√	√	√	√
Klarin (2008) (60)		√	√		√	√		√				√	√	√	√		√	√	√	√	√			
Spindler-Vesel (2007) (61)		√	√	√	√		√		√		√				√		√	√	√	√		√	√	√
Kotzampassi (2006) (14)					√			√			√	√	√	√	√	√	√		√			√		
Zarinfar (2016) (62)				√					√					√			√				√		√	
Zeng (2016) (16)				√	√			√	√		√		√	√	√	√	√	√	√	√	√	√	√	√
Klarin (2018) (63)				√					√		√			√						√				
Shimizu (2018) (19)				√					√		√		√	√		√	√		√	√	√	√	√	√
Anandaraj (2019) (64)				√					√				√	√								√	√	
Mahmoodpoor (2019) (17)				√					√		√		√	√		√	√		√	√	√	√	√	√
Habib (2020) (65)				√					√														√	
Nazari (2020) (66)				√					√				√									√	√	
Johnstone (2021) (46)				√					√				√						√			√	√	√
Tsilika (2021) (67)				√									√										√	
Oudhuis (2011) (68)					√	√			√		√			√				√						√
Li (2012) (69)					√				√									√		√				
Banupriya (2015) (70)					√		√											√		√	√			√
Besselink (2008) (71)							√	√				√	√				√				√			
Kanazawa (2005) (72)							√																	
Rayes (2002) (73)							√														√			
Rayes (2005) (74)							√																	
Rayes (2007) (75)							√																	
Tempe (1983) (76)								√				√	√								√			
Schlotterer (1987) (77)								√				√	√								√			
Heimburger (1994) (78)								√				√	√											
Bleichner (1997) (79)								√				√	√				√		√		√			
Kecskes (2003) (80)								√				√	√											
Lu (2004) (81)								√				√	√				√							
Jain (2004) (82)								√				√	√				√		√		√			
Klarin (2005) (83)								√				√	√				√		√		√			√
McNaught (2005) (84)								√				√	√				√		√		√			
Alberda (2007) (85)								√				√	√				√		√		√			
Li (2007) (86)								√				√	√								√			
Olah (2007) (87)								√				√	√				√				√			
Frohmader (2010) (88)								√				√	√				√		√		√		√	
Ferrie (2011) (89)								√				√	√				√		√		√			
Sharma (2011) (90)								√				√	√											
Cui (2013) (91)								√					√				√				√			
Tan (2013) (92)								√					√				√				√			
Wang (2013) (93)								√					√				√				√			
Lopez de Toro (2014) (94)								√					√				√		√		√			
Sanaie (2014) (95)								√					√				√		√					
Kooshki (2018) (18)									√					√			√					√	√	
Tsaousi (2019) (96)									√					√								√		
Angurana (2018) (97)									√												√			
Šrámek (2007) (98)													√											
Shinotsuka (2008) (99)													√								√			
Yan (2008) (100)													√											
Wu (2009) (101)													√											
Lata (2010) (102)													√											
Xie (2010) (103)													√								√			
Qin (2011) (104)													√								√			
Su (2011) (105)													√											
Tan (2014) (106)													√											
Cui (2009) (107)													√											
Xiong (2013) (108)													√								√			
Zhou (2013) (109)													√											
Gao (2014) (110)													√											
Ge (2014) (111)													√											
Li (2014) (112)													√											
Zhu (2014) (113)													√				√							
Chen (2015) (114)													√											
Dong (2015) (115)													√											
Han (2015) (116)													√											
Kwon (2015) (117)													√								√			
Lang (2015) (118)													√											
Peng (2015) (119)													√											
Wang (2015) (120)													√											
Ma (2016) (121)													√											
Malik (2016) (122)													√				√				√		√	
de Castro Soares (2017) (123)													√											
Li (2017) (124)													√											
Wu (2017) (125)													√											
Yang (2017) (126)													√											
Zhang (2017) (127)													√								√			
Li (2018) (128)													√											
Jin (2019) (129)													√	√									√	
Wu (2019) (130)													√	√							√			
Wan (2020) (131)													√								√			
Litton (2021) (132)													√						√		√			
Seifi (2021) (133)													√											
Wang (2021a) (134)													√											
Wang (2021b) (135)													√											
Braga (1995) (136)																	√							
Kudsk (1996) (137)																	√							
Falcão De Arruda (2004) (138)																	√		√		√			
Sun (2004) (139)																	√							
Casas (2007) (140)																	√							
Karakan (2007) (141)																	√							
Doley(2009) (142)																	√							
Moses (2009) (143)																	√						√	
Hayakawa (2012) (144)																	√				√			
Malian (2012) (145)																	√				√			
Plaudis (2012) (146)																	√							
Elke (2013) (147)																	√							
Fu (2015) (148)																	√							
Kim (2015) (149)																	√							
Fan (2016) (150)																	√							
Alberda (2018) (151)																	√							
Fazilaty (2018) (152)																	√						√	
Reiginer (2018) (153)																	√						√	
Tuncay (2018) (154)																	√							
Petrov (2006) (155)																	√							
Caparros (2001) (157)																	√						√	
Radrizzani (2006) (158)																							√	
Abdulmeguid (2007) (156)																	√						√	
Altintas (2011) (159)																							√	
Aydogmu (2012) (160)																							√	
Chung (2011) (161)																					√			
Honeycutt (2007) (162)																					√			
Kate (2020) (163)																					√			
Kumar (2013) (164)																					√			
Mallick (2018) (165)																					√			
Masjedi (2017) (166)																					√			
Mayes (2014) (167)																					√			
Olah (2002) (168)																					√			
Rammohan (2015) (169)																					√			
Shimizu (2011) (170)																					√			
Thoma (2019) (171)																					√			
Wang (2020) (172)																					√			
Xie (2013) (173)																					√			
Xie (2017) (174)																					√			
Yu (2007) (175)																					√			
Zhuang (2012) (176)																					√			

### Handling of high overlap

Given the high CCA value observed in our review, we conducted sensitivity Analyses following the approach proposed by Ma et al. to evaluate the potential impact of overlapping primary studies on the reliability of our findings. In these Analyses, duplicated primary studies were removed, and the synthesis was repeated using a non-overlapping dataset. The results of the sensitivity Analyses were then compared with those of the main analysis to assess the stability of the findings and to identify potential sources of bias.

### Data extraction

Two reviewers (YJ and DX) independently screened articles according to predefined inclusion criteria and extracted relevant data. Any discrepancies during the screening or extraction process were resolved through discussion to reach a consensus. The comprehensive workflow of study inclusion and exclusion is presented in a PRISMA-compliant flow diagram. Extracted data from eligible systematic reviews and meta-analyses included the first author’s name and publication year, number of included primary studies, total sample size, country of the corresponding author, types of included studies, data sources, probiotic strains, dosages, duration of intervention, comparator interventions, and primary and secondary outcomes. All disagreements were resolved through consultation with a third reviewer (JZ). For each meta-analysis, we extracted information on probiotic strains, formulations, and dosages. Dosages varied in terms of colony-forming units (CFU), administration frequency, and intervention duration. This variability was noted and considered when interpreting clinical outcomes, as it may influence the certainty of evidence.

### Methodological quality assessment and evidence grading

The methodological rigor of included systematic reviews and meta-analyses was evaluated using the AMSTAR 2 tool, which assesses 16 domains, including risk of bias and availability of a pre-specified protocol (21). Each domain was independently rated as “Yes,” “Partial Yes,” or “No” by two reviewers (YJ and DX). Within this framework, seven domains were identified as critical, as deficiencies in these areas could substantially affect the reliability of the review (items 2, 4, 7, 9, 11, 13, and 15). Reviews meeting all critical domains and at least eight non-critical items were classified as “high” quality. Reviews fulfilling all critical domains but not reaching the threshold in non-critical items were rated as “moderate” quality. Reviews with one critical domain deficiency were rated as “low” quality, whereas those with two or more critical deficiencies were rated as “critically low.” All eligible meta-analyses were included in the umbrella review regardless of quality. Disagreements during quality assessment were resolved through discussion with a third reviewer (JZ).

### Data synthesis and statistical analysis

Effect sizes (ESs) and corresponding 95% confidence intervals (CIs) were used to summarize outcomes. Heterogeneity was assessed using Cochran’s Q test and the I^2^ statistic, with *I^2^* > 50% or *p* < 0.1 indicating substantial heterogeneity. Pooled estimates were calculated using a random-effects model with restricted maximum likelihood (REML) estimation. To explore potential sources of heterogeneity, subgroup analyses were conducted based on predefined variables, including study sample size (<1,000 vs. ≥1,000 participants). Sensitivity Analyses were performed using a leave-one-out approach to evaluate the influence of individual studies on overall effect estimates. Small-study effects were assessed using Egger’s and Begg’s tests, as small initial studies often report inflated effect sizes relative to subsequent larger studies. Funnel plots were used to examine potential publication bias visually. When publication bias was suggested, the trim-and-fill method was applied to assess the robustness of the pooled estimates further. Publication bias analyses were not conducted when fewer than eight studies were available for a given outcome. All eligible studies were managed using EndNote 20 (Clarivate Analytics) and Microsoft Excel (Microsoft Corporation). Statistical Analyses were performed using Stata version 16 (StataCorp, College Station, TX, United States), with a significance threshold set at *p* < 0.05.

## Results

### Search process

**Figure 1:**
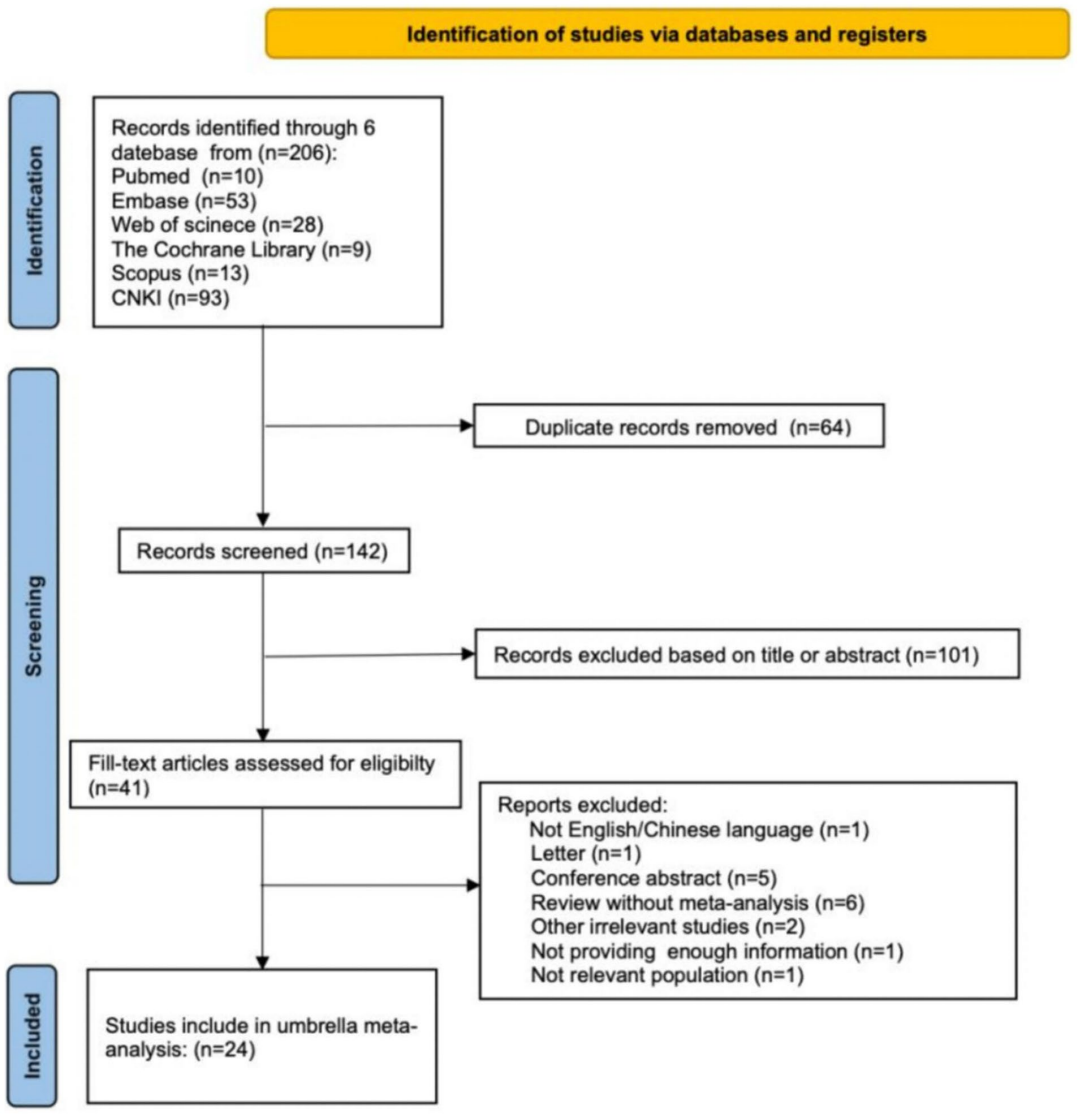
Flow diagram of the selection process.

A total of 206 articles were initially identified through searches of the 6 databases. After removing 64 duplicates, 142 records remained for screening. Of these, 101 articles were excluded following title and abstract screening for not meeting the eligibility criteria. During full-text assessment, 17 additional studies were excluded for various reasons. Ultimately, 24 published meta-analyses of RCTs met the inclusion criteria and were incorporated into this umbrella review. The detailed study selection process is presented in Figure 1.

### Characteristics of included meta-analyses and quality assessment

**Figure 2:**
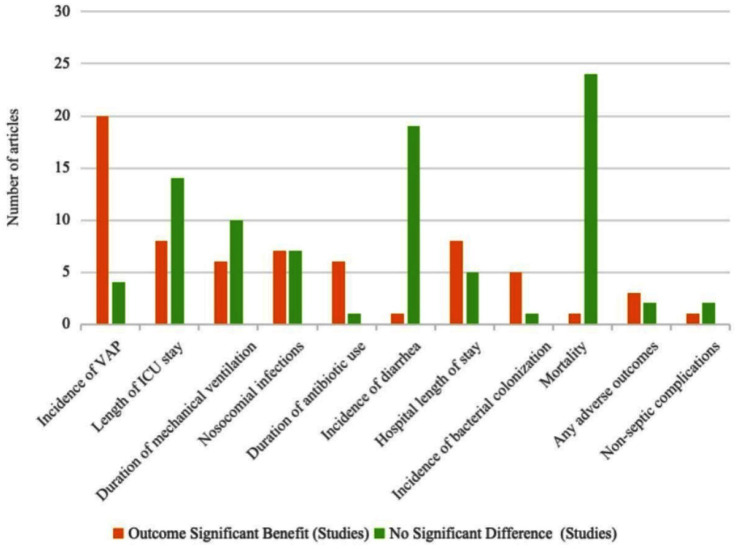
Significant benefits of probiotics versus control on clinical outcomes.

A total of 92,711 participants were included across the 24 meta-analyses. The publication years of the included studies ranged from 2010 to 2023. Seventeen studies were conducted in China, while the remaining studies were conducted in Greece (*n* = 1), India (*n* = 1), the United States (*n* = 1), Malaysia (*n* = 1), Canada (*n* = 2), and the United Kingdom (*n* = 1). The administered probiotic dosages ranged from 2 × 10^6^ to 1.8 × 10^12^ colony-forming units (CFU), with intervention durations spanning from 2 days to 6 months. Study characteristics, including details of the included RCTs, are summarized in Table 1, and the effects of probiotic supplementation on clinical outcomes are illustrated in Figure 2.

Methodological quality was assessed using the AMSTAR 2 tool in combination with a standardized data extraction form. Based on the AMSTAR 2 rating system, four meta-analyses were classified as “critically low” confidence, indicating substantial methodological limitations (9, 12, 24, 27). Twelve studies were rated as “low” confidence, suggesting potential deficiencies in study design or execution (1, 4, 13, 22, 26, 29, 31, 33, 35, 37). One study achieved a “moderate” confidence rating, reflecting adequate methodological handling but not optimal rigor (32). Finally, nine studies were rated as “high” confidence, demonstrating methodological rigor and minimal risk of bias (2, 3, 7, 23, 25, 28, 30, 34, 36). Detailed quality assessment of the included studies is presented in Tables 2, 3.

**Table 2:** Study characteristics of the included studies.

Source (First author et al., year)	No. of primary studies	No. of participants	Location	Search databases	Intervention	Dose (CFU)	Duration (day)	Primary outcomes	Other outcome(s)
Zhang et al. (2019) (22)	7	1,046	China	EMBASE, PubMed, Cochrane library	*Lactobacillus*	NR	NR	①	②
Bo et al. (2014) (8)	8	1,083	China	CENTRAL, MEDLINE, EMBASE	*Lactobacillus* spp. multi-strain Preparations:Synbiotic 2000 FORTE, Ergyphilus Bifidobacterium+*Lactobacillus*+*Streptococcus* combination	10^9^-(4×10^10^)	2–30	①②④	⑥⑦⑨⑩
Siempos et al. (2010) (9)	5	689	Greece	PubMed, Scopus, Current Contents, Cochrane	*Lactobacillus* spp. Multiple genera: *Pediococcus pentosaceus*, *Leuconostoc mesenteroides*	(2×10^9^)-(2.5×10^10^)	4–28	①	②③④⑥⑦
Cheema et al. (2022) (23)	18	4,893	Britain	Cochrane, MEDLINE, Embase, ClinicalTrials.gov and WHO International Clinical Trials Registry Platform, OATD, PQDT	*Lactobacillus* spp. *Bifidobacterium* spp. *Bacillus subtilis* *Enterococcus faecalis* *Saccharomyces boulardii* Multi-strain preparations: Synbiotic 2000 FORTE, Ergyphilus, FamiLact 2plus	NR	NR	①	②④⑤⑥⑦⑨⑩
Fan et al. (2019) (24)	14	2,036	China	PubMed, Web of Science, EMBASE, Cochrane	*Lactobacillus* spp. *Bifidobacterium* spp. *Streptococcus* *Thermophilus* *Bacillus subtilis* *Enterococcus faecalis* Combinations of *Bifidobacterium*, *Lactobacillus*, and *Enterococcus* Multi-strain preparations: Synbiotic 2000 FORTE, Ergyphilus	10^7^-10^10^	NR	①②	④⑥
Gu et al. (2012) (4)	7	1,142	China	PubMed, EMBASE	*Lactobacillus* spp. *Pediococcus* spp. *Leuconostoc* spp. Multi-strain preparations: Synbiotic 2000 FORTE	(2×10^9^)-(1×10^11^)	15–28	①	②③④⑤⑥⑦
Liu et al. (2012) (12)	12	1,546	China	PubMed, Cochrane, EMBASE	*Lactobacillus* spp. *Bifidobacterium* spp. *Pediococcus* spp. *Leuconostoc* spp. *Lactococcus* spp. *Streptococcus* spp. Multi-strain preparations: Synbiotic 2000 FORTE, Ergyphilus, Ecologic 641, Yakult BL Seichōyaku, Golden Bifid	NR	7–28	①	②③⑤⑥
Manzanares et al. (2016) (1)	30	2,972	United States	MEDLINE, Embase, CINAHL, Cochrane	*Lactobacillus* spp. *Bifidobacterium* spp. *Streptococcus* spp. *Saccharomyces* spp. *Pediococcus* spp. *Leuconostoc* spp. *Lactococcus* spp. *Bacillus* spp. *Enterococcus* spp. Multi-strain preparations: Synbiotic 2000 FORTE, Ergyphilus, Ecologic 641, Yakult BL Seichōyaku, Golden Bifid, VSL#3, Trevis™, Jinshuangqi, Symbiotic drink	(6 × 10^6^)-(9×10^11^)	5–28	③	①②④⑤⑥
Sun et al. (2022) (25)	23	5,543	China	PubMed, Embase, Cochrane	Probiotic, Synbiotic, Prebiotic	NR	14–180	①	②③④
Wang et al. (2013) (26)	5	844	China	Web of science, PubMed, Ovid, Cochrane	*Lactobacillus* spp. *Bifidobacterium* spp. Multi-strain preparations: Synbiotic 2000 FORTE *Lactobacillus* spp. + *Bifidobacterium* spp.	(2×10^9^)-(2 × 10^11^)	15–28	①	②⑤⑥⑪
Su et al. (2020) (13)	14	2,113	China	PubMed, EMBASE, Cochrane	*Lacticaseibacillus* spp. *Bifidobacterium* spp. Multi-strain preparations: Synbiotic 2000 FORTE、multispecies probiotics Yakult BL Seichoyaku Probiotic capsules/preparation	(4.2×10^8^)-10^11^	14–180	①	②④⑥⑦⑨
Petrof et al. (2020) (27)	23	2,153	Canada	EMBASE, MEDLINE, CINAHL, Cochrane	*Saccharomyces* spp. *Lactobacillus* spp. *Bifidobacterium* spp. *Streptococcus* spp. Multi-strain preparations: Lactinex, Trevisone, Proviva, Synbiotics, Synbiotic 2000 Forte, VSL#3, Jinshuangqi, Ergyphilus, Ecologic 641	(2×10^6^)-(9×10^11^)	8–28	③	①②⑤⑥
Lee et al. (2023) (28)	71	8,551	Malaya	MEDLINE, EMBASE, CENTRAL, CINAHL	*Saccharomyces* spp. *Lactobacillus* spp. *Bifidobacterium* spp. *Streptococcus* spp. *Enterococcus* spp. *Pediococcus* spp. *Leuconostoc* spp. Multi-strain preparations: Lactinex, Trevisone, Proviva, Synbiotics, Synbiotic 2000 Forte, VSL#3, Jinshuangqi, Ergyphilus, Ecologic 641	(3.06×10^5^)-(9×10^11^)	5–60	①	②③④⑤⑥⑦⑩
Ji et al. (2021) (2)	20	2,428	China	PubMed, Web of Science, EMBASE, MEDLINE, Cochrane Library, Ovid, EBSCO	*Lactobacillus* spp. *Bifidobacterium* spp. *Enterococcus* spp. *Bacillus* spp. *Pediococcus* spp. *Leuconostoc* spp. *Clostridium* spp. Multi-strain preparations: Synbiotic 2000 Forte, Ergyphilus, Golden Bifid, Medilac-S, Fenugreek seed, 4-probiotic capsule	(2×10^7^)-10^10^	1–28	①	②③⑤⑥⑦⑧⑨⑫
Chen et al. (2018) (29)	10	1,403	China	PubMed, Web of Science	*Pediococcus* spp. *Leuconostoc* spp. *Lactobacillus* spp. *Bifidobacterium* spp. *Streptococcus* spp. *Bacillus* spp. *Enterococcus* spp. Multi-strain preparations: Synbiotic 2000 FORTE, Golden Bifid, Ergyphilus	(4.2×10^8^)-10^11^	14–180	①	②④⑥⑦⑨
Batra et al. (2020) (3)	9	1,236	India	EMBASE, Pubmed, Web of Science, Cochrane	*Lactobacillus* spp. *Bifidobacterium* spp. *Pediococcus* spp. *Streptococcus* spp. Multi-strain preparations: Synbiotic 2000 FORTE, Golden Bifid, Yakult BL Seichōyaku, Medilac-S, Ergyphilus	(6×10^7^)-10^11^	14–28	①	②④⑤⑥⑦⑧
Li et al. (2021) (30)	55	7,119	China	Pubmed, Embase, Cochrane, Web of Science	*Lactobacillus* spp. *Saccharomyces* spp. *Clostridium* spp. Multi-strain preparations: Synbiotic 2000 Forte, Trevis™, VSL#3, Yakult BL Seichoyaku, Inulin, Golden Bifid, Medilac-S, Drink Simbiotic, Ergyphilus	NR	NR	③	①②③④⑤⑥⑦
Weng et al. (2017) (31)	13	1,969	China	PubMed, Embase, CENTRAL	*Lactobacillus* spp. *Bifidobacterium* spp. *Streptococcus* spp. *Bacillus* spp. *Enterococcus* spp. Multi-strain preparations: Synbiotic 2000 Forte, Ergyphilus, robiotic capsule, Golden Bifid, Yakult BL Seichoyaku, Medilac-S	(6×10^7^)-10^11^	7–180	①	②④⑤⑥⑦
Wang et al. (2022) (32)	25	5,049	China	PubMed, Web of Science, Cochrane, CNKI, WHO Global Index Medicus, CBM	*Lactobacillus* spp. *Bifidobacterium* spp. *Streptococcus* spp. *Bacillus* spp. *Saccharomyces* spp. *Enterococcus* spp. Multi-strain preparations: Synbiotic 2000 Forte, Synbiotic drink, Medilac-S, B. breve strain, *L. casei* strain + galactooligosaccharides, *L. acidophilus* La5, *B. lactis* Bb-12, *S. thermophilus*, *L. bulgaricus*	NR	5–60	②	①③⑥⑫
Zhao et al. (2021) (33)	15	2,039	China	PubMed, Medline, EMBASE, Cochrane, CNKI	*Lactobacillus* spp. *Bifidobacterium* spp. *Streptococcus* spp. Multi-strain preparations: Synbiotic 2000 FORTE, Yakult BL Seichoyaku, Lactobacillus + *Bifidobacterium* + *Streptococcus Thermophilus*, *Bacillus subtilis* + *Enterococcus faecalis*, *Lactobacillus* + *Bifidobacterium* + *Streptococcus thermophilus*	10^8^-(8×10^10^)	2–28	①	②⑤⑥⑦⑨⑫
Sharif et al. (2022) (34)	65	8,483	Canada	MEDLINE, EMBASE, CENTRAL, ClinicalTrials.gov, WHO International Clinical Trials Registry Platform, Latin-American, Caribbean System on Health Sciences	*Lactobacillus* spp. *Bifidobacterium* spp. *Saccharomyces* spp. *Clostridium* spp. Multi-strain preparations: Synbiotic 2000 Forte, Trevis™, VSL#3, Golden Bifid, Ecologic 6416, Ergyphilus, Prowel	NR	NR	①③	②④⑤⑥
Lan et al. (2022) (35)	15	4,561	China	PubMed, Web of Science, MEDLINE, Embase, Cochrane, ClinicalTrials.gov databases	*Lactobacillus* spp. *Bifidobacterium* spp. *Streptococcus* spp. *Bacillus* spp. *Enterococcus* spp. Multi-strain preparations: Medilac-S, fenugreek seed, *Lactobacillus* + *Bifidobacterium* ± *Streptococcus*/*Saccharomyces*/*Pediococcus*	NR	NR	①	②⑤⑥⑦
Li et al. (2022) (36)	31	8,339	China	Cochrane, Embase, Pubmed, Web of Science	*Lactobacillus* spp. *Bifidobacterium* spp. *Streptococcus* spp. *Bacillus* spp. Multi-strain preparations: Synbiotic 2000 Forte, VSL#3, Yakult, Golden Bifid, Medilac-S, Ergyphilus, Lactocare	(6×10^7^)-(9×10^11^)	NR	①	②③④⑤⑥⑦
Song et al. (2022) (37)	15	4,693	China	PubMed, Embase, Scopus, Cochrane	*Lactobacillus* spp. Multi-strain preparations: Yakult BL Seichoyaku, Synbiotic preparation, Synbiotic formula, *Lactobacillus* + *Bifidobacterium* + *Streptococcus*, *Bacillus* + *Enterococcus*, *Pediococcus* + *Leuconostoc* + *Lactobacillus*	NR	NR	①②	④⑥⑦⑧

OATD, Open Access Theses and Dissertations; PQDT, ProQuest Dissertations and Theses Global; CNKI, China Knowledge Resource Integrated Database; CBM: Chinese Biomedicine Database; NR, Not reported; VAP: Ventilator-associated pneumonia; Outcome indicators: ① Incidence of VAP; ② Mortality; ③ Nosocomial infections (including bloodstream infections, catheter-related bloodstream infection, urinary tract infection); ④ Incidence of diarrhea; ⑤ Hospital length of stay; ⑥ Length of ICU stay; ⑦ Duration of mechanical ventilation; ⑧ Incidence of bacterial colonization; ⑨ Duration of antibiotic use; ⑩ Any adverse outcomes of the probiotics; ⑪ Etiology studies; ⑫ Non-septic complications.

**Table 3:** AMSTAR 2 score for included articles.

Source (First author et al., year)	Q1	Q2^a^	Q3	Q4^a^	Q5	Q6	Q7^a^	Q8	Q9^a^	Q10	Q11^a^	Q12	Q13^a^	Q14	Q15^a^	Q16	Overall confidence
Zhang et al. (2019) (22)	Y	N	Y	PY	Y	Y	PY	PY	Y	N	Y	Y	Y	Y	Y	N	Low
Bo et al. (2014) (8)	Y	Y	Y	Y	Y	Y	PY	Y	Y	Y	Y	Y	Y	Y	Y	Y	High
Siempos et al. (2010) (9)	Y	N	Y	PY	Y	Y	Y	PY	Y	N	Y	Y	Y	Y	N	Y	Critically low
Cheema et al. (2022) (23)	Y	Y	Y	PY	Y	Y	Y	PY	Y	N	Y	Y	Y	Y	Y	Y	High
Fan et al. (2019) (24)	Y	PY	Y	PY	Y	Y	PY	PY	Y	N	Y	N	N	Y	N	Y	Critically low
Gu et al. (2012) (4)	Y	N	Y	PY	Y	Y	Y	PY	Y	N	Y	Y	Y	Y	Y	Y	Low
Liu et al. (2012) (12)	Y	N	Y	PY	Y	Y	Y	PY	Y	N	Y	N	N	Y	Y	Y	Critically low
Manzanares et al. (2016) (1)	Y	PY	Y	PY	Y	Y	Y	Y	Y	N	Y	Y	N	Y	Y	Y	Low
Sun et al. (2022) (25)	Y	PY	Y	PY	Y	Y	Y	PY	Y	N	Y	Y	Y	Y	Y	Y	High
Wang et al. (2013) (26)	Y	PY	Y	PY	Y	Y	PY	PY	Y	N	Y	N	N	Y	Y	Y	Low
Su et al. (2020) (13)	Y	N	Y	PY	Y	Y	Y	Y	Y	N	Y	Y	Y	Y	Y	Y	Low
Petrof et al. (2020) (27)	Y	N	Y	PY	Y	Y	Y	Y	Y	N	Y	N	Y	Y	N	Y	Critically low
Lee et al. (2023) (28)	Y	Y	Y	PY	Y	Y	Y	Y	Y	Y	Y	Y	Y	Y	Y	Y	High
Ji et al. (2021) (2)	Y	Y	Y	Y	Y	Y	Y	PY	Y	N	Y	Y	Y	Y	Y	Y	High
Chen et al. (2018) (29)	Y	N	Y	PY	Y	Y	PY	PY	Y	N	Y	Y	Y	Y	Y	Y	Low
Batra et al. (2020) (3)	Y	Y	Y	PY	Y	Y	Y	Y	Y	N	Y	Y	Y	Y	Y	Y	High
Li et al. (2021) (30)	Y	Y	Y	PY	Y	Y	Y	PY	Y	N	Y	Y	Y	Y	Y	Y	High
Weng et al. (2017) (31)	Y	PY	Y	PY	Y	Y	Y	Y	Y	N	Y	Y	N	N	Y	Y	Low
Wang et al. (2022) (32)	Y	PY	Y	PY	Y	Y	Y	PY	Y	N	Y	N	Y	Y	Y	Y	Moderate
Zhao et al. (2021) (33)	Y	PY	Y	PY	Y	Y	Y	PY	Y	N	Y	N	N	Y	Y	Y	Low
Sharif et al. (2022) (34)	Y	Y	Y	PY	Y	Y	Y	Y	Y	N	Y	Y	Y	Y	Y	Y	High
Lan et al. (2022) (35)	Y	Y	Y	PY	Y	Y	Y	Y	Y	N	Y	Y	N	N	Y	Y	Low
Li et al. (2022) (36)	Y	Y	Y	PY	Y	Y	Y	PY	Y	N	Y	Y	Y	Y	Y	Y	High
Song et al. (2022) (37)	Y	Y	Y	PY	Y	Y	Y	PY	Y	N	Y	N	N	Y	Y	Y	Low

Q1: Did the research questions and inclusion criteria for the review include the components of PICO?

Q2: Did the report of the review contain an explicit statement that the review methods were established prior to the conduct of the review and did the report justify any significant deviations from the protocol?

Q3: Did the review authors explain their selection of the study designs for inclusion in the review?

Q4: Did the review authors use a comprehensive literature search strategy?

Q5: Did the review authors perform study selection in duplicate?

Q6: Did the review authors perform data extraction in duplicate?

Q7: Did the review authors provide a list of excluded studies and justify the exclusions?

Q8: Did the review authors describe the included studies in adequate detail?

Q9: Did the review authors use a satisfactory technique for assessing the risk of bias (RoB) in individual studies that were included in the review?

Q10: Did the review authors report on the sources of funding for the studies included in the review?

Q11: If meta-analysis was performed did the review authors use appropriate methods for statistical combination of results?

Q12: If meta-analysis was performed, did the review authors assess the potential impact of RoB in individual studies on the results of the meta-analysis or other evidence synthesis?

Q13: Did the review authors account for RoB in individual studies when interpreting/discussing the results of the review?

Q14: Did the review authors provide a satisfactory explanation for, and discussion of, any heterogeneity observed in the results of the review?

Q15: If they performed quantitative synthesis did the review authors carry out an adequate investigation of publication bias (small study bias) and discuss its likely impact on the results of the review?

Q16: Did the review authors report any potential sources of conflict of interest, including any funding they received for conducting the review?

a The AMSTAR-2 research team selected 7 items that were key to the making of systematic review and the validity of its results, namely items 2, 4, 7, 9, 11, 13 and 15. High: none or only one non-key item is not met; Moderate: more than one non-key item is not met; Low: 1 key item does not meet and with or without non-key item does not meet; Critically low: more than 1 key item is not met, with or without non-key items are not met.

### Umbrella review of different outcomes

#### Effect of probiotic supplementation on ventilator-associated pneumonia and nosocomial infections

A total of 24 RCTs were included. See Table 1 for details. Among these, three studies reported that probiotic supplementation did not appear to meaningfully affect the risk of VAP in critically ill patients, whereas 21 studies suggested a potential reduction in VAP incidence following probiotic intervention. The geographic distribution of these studies was heterogeneous: a total of 6 studies were conducted in Greece, India, France, the United States, Malaysia, and the United Kingdom; 2 studies were conducted in Canada; and the remaining 17 studies were conducted in China. Probiotic doses varied across different intervention protocols, and some studies did not report this key information. Treatment duration ranged from 2 days to 6 months, depending on the probiotic strain and study protocol.

**Figure 3:**
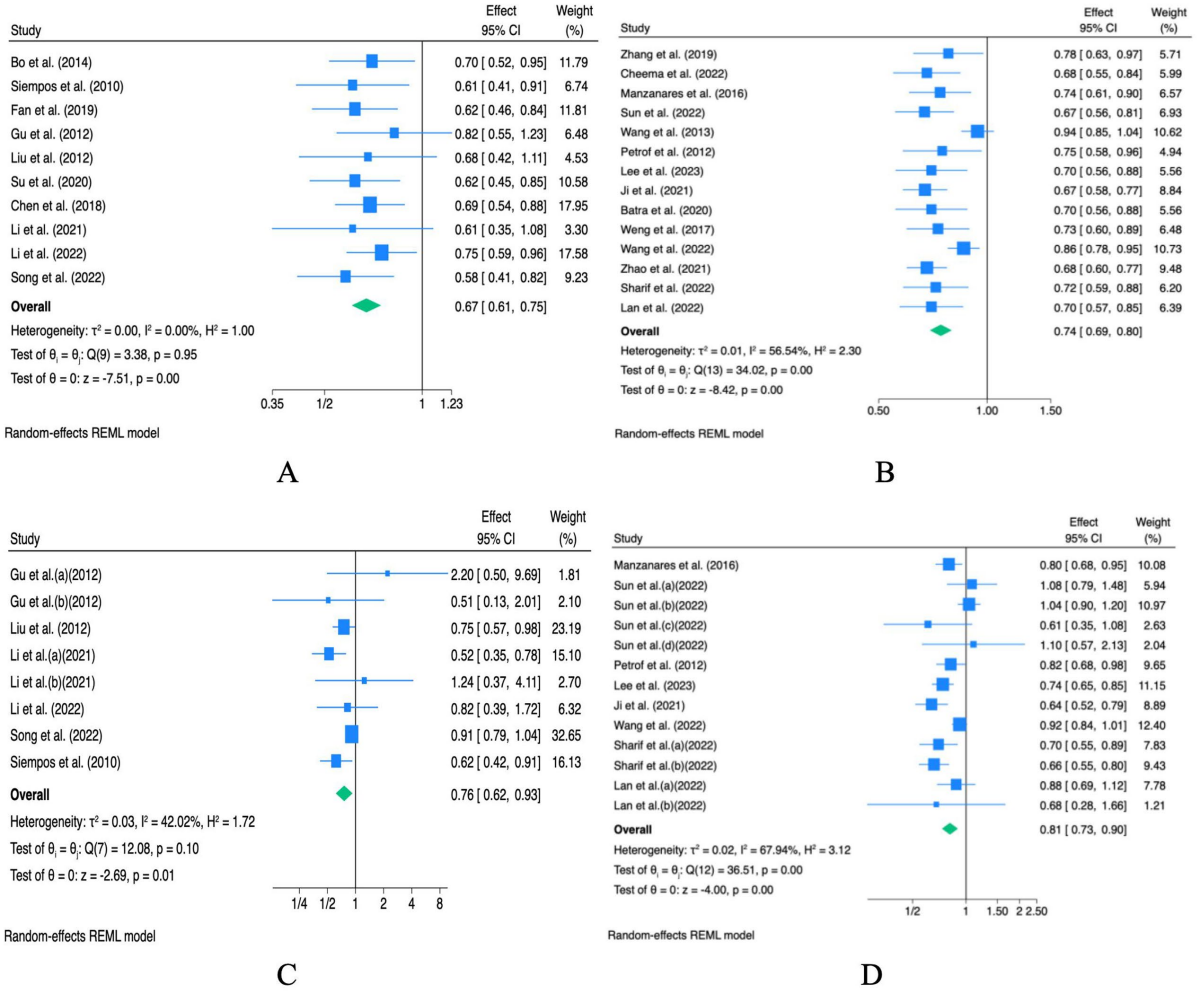
Forest plots indicating ESs and 95% CIs regarding the effects of probiotic supplementation on the incidence of VAP (ORs **(A)** and RRs **(B)**) and the incidence of nosocomial infections [ORs **(C)** and RRs **(D)**].

Pooled analysis based on odds ratios (ORs) from 10 meta-analyses indicated that probiotic intervention was associated with a reduced incidence of VAP in critically ill patients (OR = 0.67, 95% CI 0.61–0.75, *p* < 0.001; I^2^ = 0.0%, *p* = 0.95; Figure 3A). Sensitivity analysis suggested that the overall OR was generally stable, although individual studies had some influence (Supplementary Figure 1A). Subsequent subgroup analysis by study sample size revealed no significant differences between subgroups (Supplementary Figure 1B). Begg’s and Egger’s tests did not detect evidence of small-study effects (*p* = 1.000 and 0.625, respectively; Supplementary Figure 1C), and visual inspection of the funnel plot suggested apparent publication bias (Supplementary Figure 1C). Thus, the trim-and-fill analysis imputed studies (Supplementary Figure 1D).

Pooled analysis based on risk ratios (RRs) from 14 studies suggested that probiotic intervention may reduce the incidence of VAP in critically ill patients (RR = 0.74, 95% CI 0.69–0.80, *p* < 0.001; Figure 3B). Sensitivity analysis indicated that the overall effect estimate was relatively stable. However, study-specific factors could contribute to variability (Supplementary Figure 2A). Significant heterogeneity was observed among studies (*I^2^* = 56.54%, *p <* 0.001), prompting subgroup analysis, which suggested that sample size might partly explain the observed heterogeneity (Supplementary Figure 2B). Notably, in the subgroup of studies with sample sizes <1,000, probiotics did not appear to reduce VAP incidence meaningfully. Visual inspection of the funnel plot indicated potential publication bias (Supplementary Figure 2C), and Begg’s and Egger’s tests revealed evidence of small-study effects (*p* = 0.742 and 0.047, respectively; Supplementary Figure 2C). Thus, the trim-and-fill analysis imputed studies (Supplementary Figure 2D).

A pooled analysis of eight ORs from 6 studies (Figure 3C) and 13 RRs from eight studies (Figure 3D) indicated that probiotics may reduce the incidence of hospital-acquired infections (OR = 0.76; 95% CI: 0.62–0.93, *p =* 0.01, *I^2^* = 42.02%, *p* = 0.010; RR = 0.81; 95% CI: 0.73–0.90, *p* < 0.001; *I^2^* = 67.94%, *p* < 0.001). Significant heterogeneity was observed among studies. To explore the source of heterogeneity, a preplanned subgroup analysis stratified by sample size was planned, but it could not be performed because all studies had sample sizes ≥1,000. Furthermore, Sensitivity analysis suggested limited robustness of the pooled OR, whereas the pooled RR appeared stable (Supplementary Figures 3A, 4A). Visual inspection of the funnel plots did not reveal any publication bias (Supplementary Figures 3C, 4C). Additionally, Begg’s and Egger’s tests indicated that small-study effects for both OR and RR were not statistically significant (*p* > 0.05; Supplementary Figures 3B, 4B).

#### Effect of probiotic supplementation on ICU and total hospital length of stay

**Figure 4:**
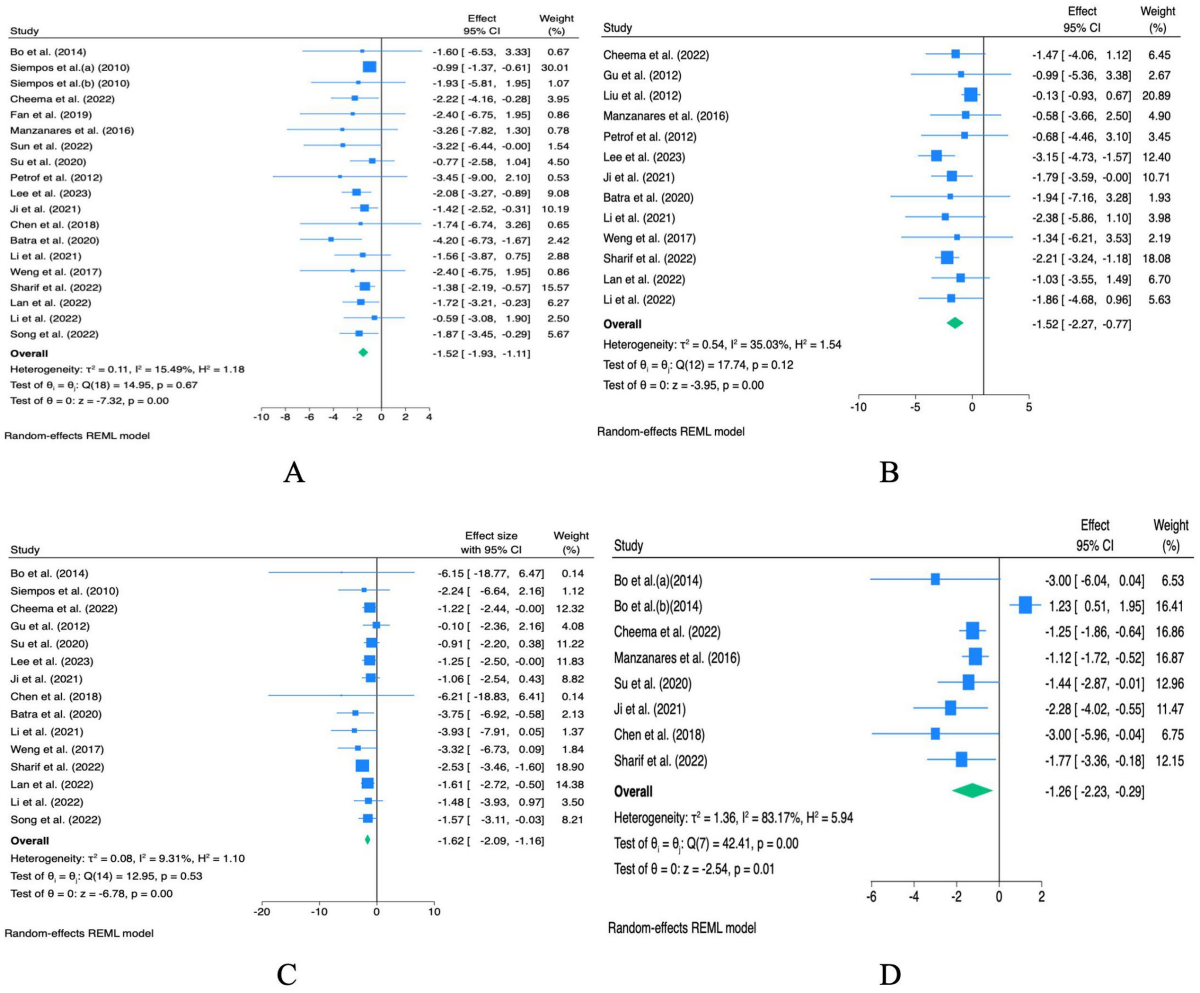
Forest plots indicating ESs and 95% CIs regarding the effects of probiotic supplementation on the duration of ICU length of stay [WMDs **(A)**], hospital length of stay [WMDs **(B)**], duration of MV [WMDs **(C)**], duration of antibiotic use [WMDs **(D)**].

Weighted mean differences (WMDs) analysis of 19 effect sizes (ES) from 18 studies demonstrated that probiotic supplementation may reduce ICU length of stay (ES WMD = -1.52; 95% CI: −1.93 to −1.11; *p* < 0.001; *I^2^* = 15.49%, *p* = 0.67; Figure 4A). Sensitivity analysis indicated that the pooled effect was somewhat influenced by individual studies, suggesting limited robustness of the overall result (Supplementary Figure 5A). Begg’s test did not reveal significant small-study effects (*p* = 0.441; Supplementary Figure 5B), whereas Egger’s test indicated potential small-study effect (*p* = 0.009; Supplementary Figure 5B). Visual inspection of the funnel plot suggested potential publication bias (Supplementary Figure 5C). Thus, the trim-and-fill analysis imputed studies (Supplementary Figure 5D).

Similarly, meta-analysis of 13 studies appeared to show that probiotics may shorten total hospital stay (ES WMD = -1.52; 95% CI: −2.27 to −0.77; *p* < 0.001; *I^2^* = 32.03%, *p* = 0.12; Figure 4B). Considerable heterogeneity was observed across the studies. To explore potential sources of heterogeneity, subgroup analysis by sample size was not feasible because all studies had sample sizes ≥1,000. Sensitivity analysis demonstrated that the pooled effect was sensitive to certain individual studies, indicating limited robustness (Supplementary Figure 6A). Both Egger’s and Begg’s tests did not detect small-study effects (*p* = 0.937 and 0.675, respectively; Supplementary Figure 6B). Visual inspection of the funnel plot suggested potential publication bias (Supplementary Figure 6C). Thus, the trim-and-fill analysis imputed studies (Supplementary Figure 6D).

#### Effect of probiotic supplementation on duration of antibiotic use and incidence of diarrhea

A pooled analysis of 13 studies demonstrated that probiotic supplementation significantly reduced the duration of antibiotic use (WMD = -1.26 days; 95% CI: −2.23 to −0.29, *p* = 0.01; *I*^2^ = 83.17%, *p* < 0.001; Figure 4D). Significant heterogeneity was observed. Subgroup analysis was not feasible due to study sample size constraints. Sensitivity analysis indicated that no single study substantially influenced the pooled effect (Supplementary Figure 7A). Egger’s test indicated the presence of small-study effects (*p* = 0.043), whereas Begg’s test did not detect significant small-study effects (*p* = 0.720; Supplementary Figure 7B). Visual inspection of the funnel plot suggested potential publication bias (Supplementary Figure 7C). Thus, the trim-and-fill analysis imputed studies in order to correct the WMD (Supplementary Figure 8D).

**Figure 5:**
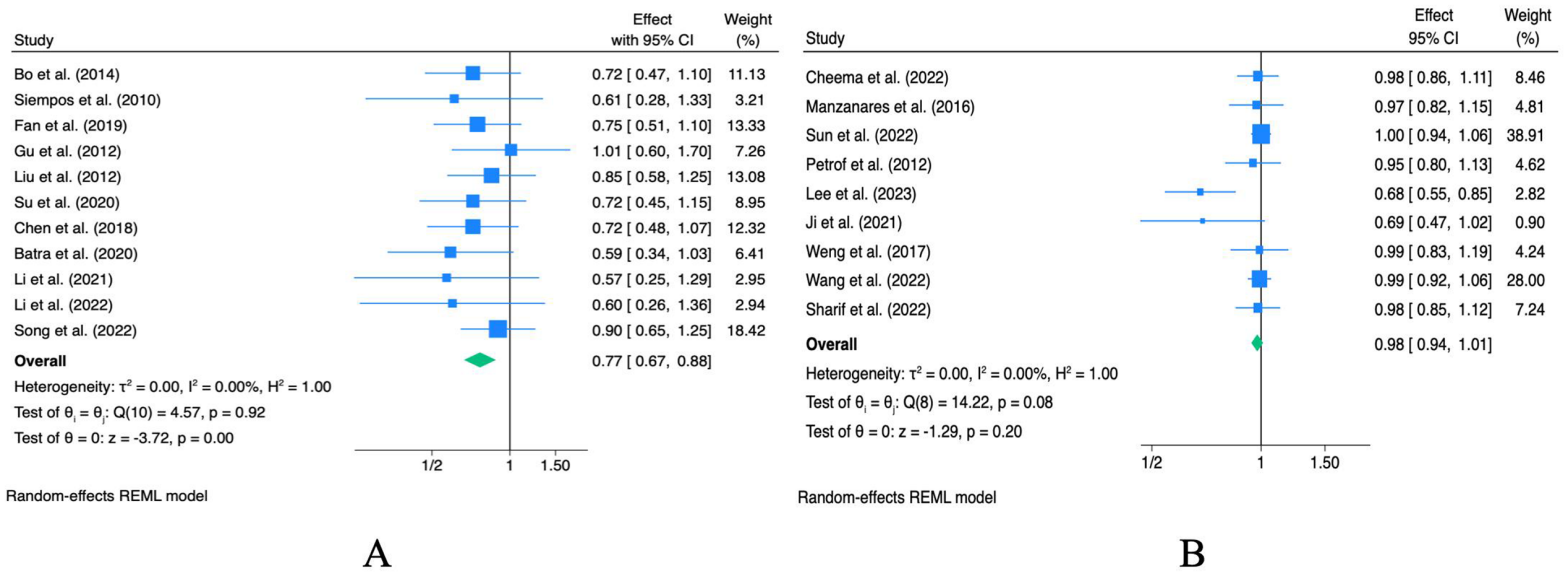
Forest plots indicating ESs and 95% CIs regarding the effects of probiotic supplementation on the incidence of diarrhea [ORs **(A)** and RRs **(B)**].

Regarding diarrhea incidence, pooled OR analysis from 11 studies showed that probiotic supplementation may reduce the risk of diarrhea (OR = 0.77; 95% CI: 0.67–0.88, *p* < 0.001; *I*^2^ = 0.0%, *p* = 0.92; Figure 5A). Sensitivity analysis showed stability of the OR across studies (Supplementary Figure 8A). Begger’s test indicated the presence of small-study effects (*p* = 0.041), whereas Egger’s test did not detect significant small-study effects (*p* = 0.181; Supplementary Figure 8B). Visual inspection of the funnel plots did not reveal any publication bias (Supplementary Figure 8C).

In contrast, RR-based analysis of nine studies did not indicate a meaningful reduction in diarrhea incidence (RR = 0.98; 95% CI: 0.94–1.01, *p* = 0.20; *I^2^* = 0.0%, *p* = 0.08; Figure 5B). Sensitivity analysis confirmed the stability of the effect estimate, and the funnel plot and statistical tests did not provide evidence of significant publication bias, Begg’s and Egger’s tests indicated significant small-study effects (Supplementary Figures 9A–C).

#### Effect of probiotic supplementation on mechanical ventilation duration and mortality

Based on the WMD analysis, the pooled results of 15 studies with 15 ES showed that probiotics shortened the duration of mechanical ventilation (ES WMD = -1.62; 95% CI -2.09 to −1.16, *p* < 0.001; *I^2^* = 9.31%, *p* = 0.53; Figure 4C). Sensitivity analysis indicated that was any single study did not influence the overall pooled result (Supplementary Figure 10A). Begg’s and Egger’s tests did not detect significant small-study effects (*p* > 0.05; Supplementary Figure 10B), but the funnel plot suggested the presence of publication bias (Supplementary Figure 10C). Thus, the trim-and-fill analysis imputed studies (Supplementary Figure 10D).

**Figure 6:**
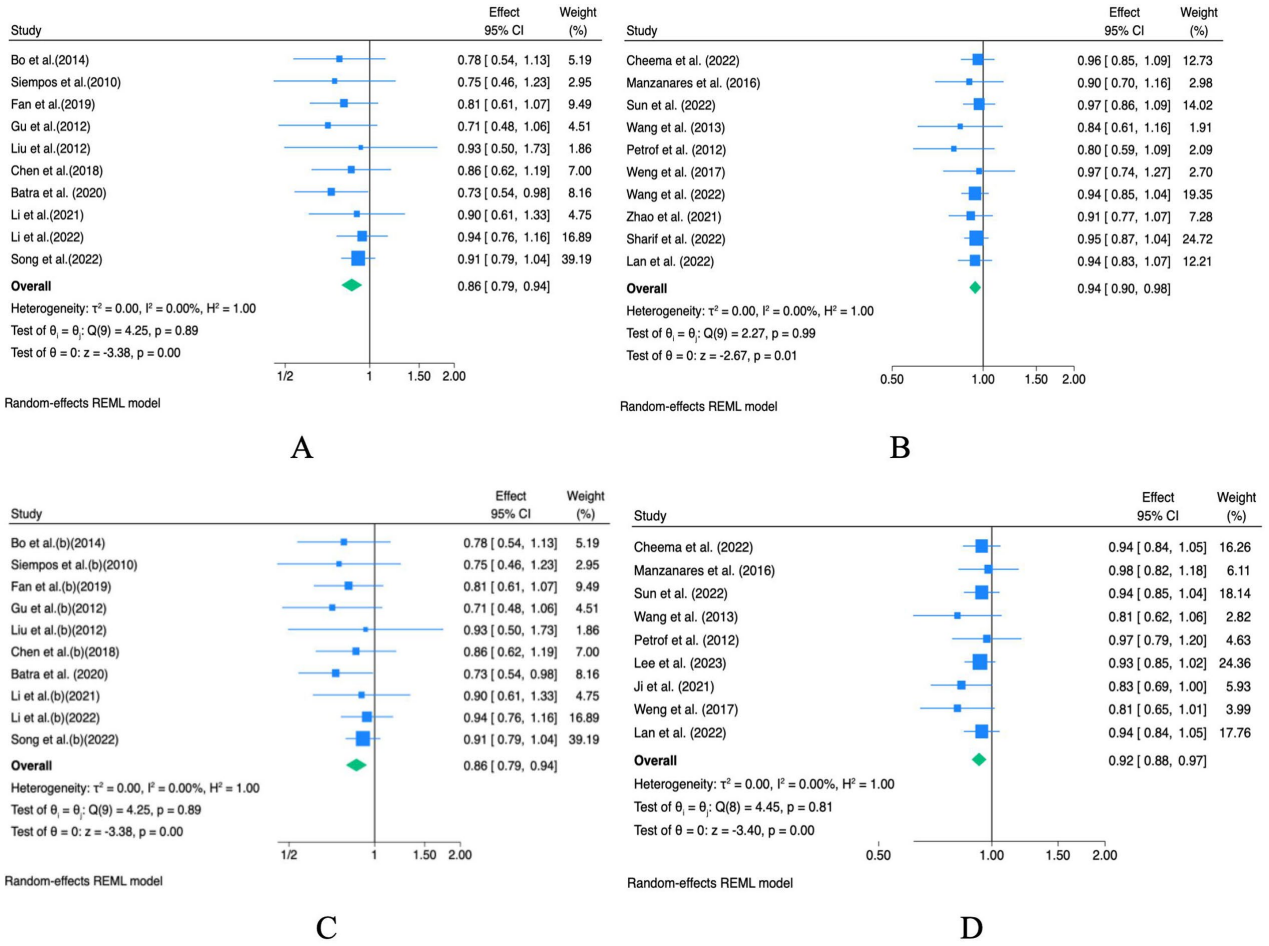
Forest plots indicating ESs and 95% CIs regarding the effects of probiotic supplementation on ICU mortality [ORs **(A)** and RRs **(B)**] and hospital mortality [ORs **(C)** and RRs **(D)**].

According to the OR analysis, probiotic supplementation was associated with a reduction in ICU mortality (OR = 0.86; 95% CI 0.79–0.94, *p <* 0.001, *I^2^* = 0.0%, *p* = 0.89; 10 meta-analyses; Figure 6A), with RR analysis yielding consistent results (RR = 0.94; 95% CI: 0.90–0.98, *p* = 0.01; I^2^ = 0.0%, *p* = 0.99; 10 meta-analyses; Figure 6B). No heterogeneity was observed among studies. Sensitivity analysis showed that excluding any single study did not affect the overall estimates of OR (Supplementary Figure 11A) and RR (Supplementary Figure 12B). Begg’s and Egger’s tests detected small-study effects for the overall OR (*p* < 0.05; Supplementary Figure 11B) but not for the overall RR (*p* > 0.05; Supplementary Figure 12B). Additionally, Visual inspection of the funnel plots did not suggest publication bias for the OR-based analyses, whereas possible publication bias was observed in the RR-based analyses (Supplementary Figures 11C, 12C). Thus, the trim-and-fill analysis imputed studies (Supplementary Figure 12D).

Regarding hospital mortality, OR-based meta-analysis did not show a statistically significant reduction (OR = 0.86; 95% CI: 0.79–0.94, *p* < 0.001; *I^2^* = 0.0%, *p* = 0.89; 10 studies; Figure 6C), whereas RR-based meta-analysis suggested a modest reduction (RR = 0.92; 95% CI 0.88–0.97, *p* < 0.001; *I^2^* = 0.0%, *p* = 0.81; 10 studies; Figure 6D). No heterogeneity was observed among studies. Sensitivity analysis demonstrated that no single study substantially altered the overall OR (Supplementary Figure 13A) or RR (Supplementary Figure 14A). Both Egger’s and Begg’s tests did not detect small-study effects for either OR or RR (*p* > 0.05; Supplementary Figures 13B, 14B). However, no evidence of publication bias was detected based on either RR or OR analyses, as indicated by the funnel plots and corresponding statistical tests (Supplementary Figures 13C, 14C).

## Discussion

This umbrella review included 24 meta-analyses evaluating the effects of probiotics on clinical outcomes in mechanically ventilated critically ill patients. Infection remains a major contributor to mortality in these patients, with ICU and in-hospital mortality rates nearly doubling compared to non-infected individuals (43). The efficacy of probiotics in such populations may be limited by severe comorbidities, prior antibiotic exposure, and impaired gastrointestinal function (26). Moreover, some studies suggest that outcomes can be influenced by the inclusion of trauma or immunocompromised patients, indicating that probiotics may not be suitable for individuals with severely compromised immunity (6, 9). Conversely, other evidence highlights potential benefits of probiotics in trauma and surgical patients, as well as in individuals at high risk for hospital-acquired pneumonia (6, 44).

The results suggest that probiotic supplementation may be associated with reductions in VAP, hospital-acquired infections, ICU and overall hospital stay, ICU and hospital mortality, duration of mechanical ventilation, antibiotic use, and diarrhea. Subgroup indicated that probiotics could be particularly beneficial in reducing VAP, hospital-acquired infections, ICU stay, hospital stay, ICU mortality, duration of mechanical ventilation, and antibiotic use when based on pooled OR and RR estimates. Significant heterogeneity was observed across studies, and subgroup Analyses suggested that sample size and study characteristics could be potential sources, highlighting that smaller studies tended to show larger effect sizes, which may limit the generalizability of findings. Significant effects on diarrhea were observed only in Analyses using OR data, whereas hospital mortality was significant only in pooled RR data. We note that this discrepancy may be due to the inherent statistical properties of ORs and RRs. ORs tend to overestimate effect sizes when event rates are high, whereas RRs directly reflect absolute risk changes and are more clinically interpretable. Additionally, variability in diarrhea definitions across studies, patient baseline conditions, and disease severity likely contributed to these differences. Similarly, heterogeneity in patient characteristics, probiotic strains, dose, administration route, intervention duration, and mortality definitions may explain the differences in hospital mortality results between OR and RR Analyses. Analyses using WMD demonstrated reductions in overall hospital stay, ICU stay, and mechanical ventilation duration, although the magnitude of effects varied across studies and should be interpreted with caution given the heterogeneity and variable quality of included meta-analyses.

In critically ill patients, the normal microbiota of the upper respiratory tract, stomach, and intestines is often replaced by the overgrowth of potential pathogens. This dysbiosis increases the risk of sepsis, multiple organ dysfunction syndrome, ventilator-associated pneumonia, and hospital-acquired infections, thereby elevating morbidity and mortality (38). Probiotics, as a non-antibiotic intervention, have attracted increasing attention due to their multiple biological functions. On the one hand, probiotics can modulate intracellular signaling pathways to exert anti-apoptotic effects, thereby reducing intestinal epithelial cell injury (39). On the other hand, they downregulate pro-inflammatory cytokines such as TNF-*α* and IL-6 while upregulating anti-inflammatory cytokines such as IL-10, effectively attenuating systemic inflammation (40). Moreover, probiotics strengthen gastrointestinal barrier integrity by enhancing the expression of tight junction proteins such as occludin and claudin, which reduces intestinal permeability and prevents bacterial and toxin translocation (41, 42). However, In trauma or immunocompromised patients, probiotics may pose risks due to impaired gut barrier and immune defenses. Translocation of probiotic bacteria into the bloodstream can cause bacteremia or systemic infection. Immune dysfunction may also prevent effective control of even low-virulence strains, increasing infection risk. Therefore, probiotics should be used cautiously in these populations (53). Collectively, these mechanisms may explain the potential benefits of probiotics in infection prevention, inflammation control, and maintenance of gut homeostasis in critically ill patients.

VAP is a common complication among mechanically ventilated patients, and probiotics may reduce its incidence through multiple mechanisms. First, probiotics competitively inhibit the colonization of pathogens in the oropharynx and gastrointestinal tract, thereby decreasing the risk of multidrug-resistant Gram-negative infections (45). Second, they help maintain microbial balance and enhance intestinal barrier integrity, preventing bacterial translocation (46). In addition, probiotics modulate host immune responses and strengthen defense mechanisms, further reducing infectious complications (47). Evidence from specific strains suggests that *Lactobacillus rhamnosus* may help prevent VAP and shorten hospital stay (47), whereas combinations such as *Bifidobacterium longum*, *Lactobacillus bulgaricus*, and *Streptococcus thermophilus* appear most effective (41). However, these findings are based on a limited number of high-quality meta-analyses.

Although most studies suggest beneficial effects of probiotics on clinical outcomes, some have reported no significant improvements. Potential reasons include heterogeneity in baseline conditions among critically ill patients, such as heart failure, respiratory failure, or severe pneumonia, which may attenuate probiotic efficacy (13, 28, 37). Variations in probiotic strains, dosages, administration routes, and intervention duration further contribute to inconsistent outcomes, while inconsistent definitions of endpoints, such as VAP, hospital-acquired infection, or diarrhea, also play a role (2, 3, 13, 30, 32). Sensitivity Analyses generally indicated that results were not driven by any single study. However, diarrhea outcomes were particularly affected by definitional inconsistencies—for example, differences in criteria such as frequency, stool characteristics, or combined indicators—with many studies failing to distinguish antibiotic-associated diarrhea, thus limiting reliability (29, 36). In specific populations, such as those with intestinal ischemia, severe pancreatitis, or prolonged high-dose use, probiotics have even been associated with increased risks (48, 49). Overall, the differences in patient characteristics, intervention protocols, and outcome definitions all contributed to the clinical efficacy of probiotics in critically ill patients.

The choice of probiotic strain is therefore critical. Only a few studies have examined strain-specific effects. Meta-analyses suggest that the combination of *B. longum*, *L. bulgaricus*, and *S. thermophilus* is particularly effective for VAP prevention (23), while Synbiotic 2000FORTE appears superior in reducing ICU and hospital mortality as well as VAP incidence (8, 13, 23). Distinct strain-specific effects were observed: *L. rhamnosus* was associated with the lowest risk of diarrhea, whereas *Lacticaseibacillus casei* carried the highest risk (23). *Lactobacillus plantarum*, alone or in combination, significantly reduced infections (1). Some evidence indicates that probiotics alone may be more effective than synbiotic preparations (1), while other studies support greater efficacy of combined formulations (9). These findings underscore the strain-dependent effects of probiotics, which contribute to heterogeneity and limit the precision of subgroup Analyses and clinical recommendations.

The strengths of this umbrella review lie in its comprehensive synthesis of 24 meta-analyses including more than 92,000 critically ill patients, incorporation of the most recent evidence, and evaluation across multiple clinical outcomes. Nevertheless, limitations include substantial heterogeneity in populations, disease types, probiotic strains, doses, and treatment durations; variable methodological quality of included meta-analyses; potential publication bias and small-study effects; and uncertain safety and efficacy in specific patient subgroups. The limited number of high-quality meta-analyses further constrains the strength and generalizability of the conclusions.

## Conclusion

This study demonstrates that probiotic supplementation may reduce VAP, hospital-acquired infections, ICU and overall hospital stay, mortality, duration of mechanical ventilation, antibiotic use, and diarrhea, suggesting its role as an effective adjunctive therapy for mechanically ventilated patients. However, strain heterogeneity, lack of comparative evaluation, and patient-specific factors such as age, comorbidities, immune status, and baseline gut microbiota, as well as the limited number of high-quality meta-analyses, constrain the strength of these conclusions. At present, no specific probiotic regimen can be recommended, and further large, rigorously designed trials are warranted to provide stronger evidence.

## Data Availability

The original contributions presented in the study are included in the article/supplementary material, further inquiries can be directed to the corresponding author.
